# Hybrid Clouds for Data-Intensive, 5G-Enabled IoT Applications: An Overview, Key Issues and Relevant Architecture

**DOI:** 10.3390/s19163591

**Published:** 2019-08-17

**Authors:** Panagiotis Trakadas, Nikolaos Nomikos, Emmanouel T. Michailidis, Theodore Zahariadis, Federico M. Facca, David Breitgand, Stamatia Rizou, Xavi Masip, Panagiotis Gkonis

**Affiliations:** 1 General Department, National and Kapodistrian University of Athens, 34400 Psahna, Greece; 2 Department of Information and Communication Systems Engineering, University of the Aegean, 83200 Samos, Greece; 3 Department of Electrical and Electronic Engineering, University of West Attica, 12244 Aigaleo, Greece; 4 Martel Lab, Martel GMBH, 6900 Lugano, Switzerland; 5 IBM Israel, Science and Technology Ltd, Haifa 3498825, Israel; 6 Singular Logic S.A., 14564 Kifisia, Greece; 7 CRAAX, Universitat Politecnica de Catalunya, 08800 Vilanova i la Geltru, Spain

**Keywords:** hybrid clouds, PaaS, data pipelines, IoT applications, cloud native solutions, cloud-to-fog infrastructure

## Abstract

Hybrid cloud multi-access edge computing (MEC) deployments have been proposed as efficient means to support Internet of Things (IoT) applications, relying on a plethora of nodes and data. In this paper, an overview on the area of hybrid clouds considering relevant research areas is given, providing technologies and mechanisms for the formation of such MEC deployments, as well as emphasizing several key issues that should be tackled by novel approaches, especially under the 5G paradigm. Furthermore, a decentralized hybrid cloud MEC architecture, resulting in a Platform-as-a-Service (PaaS) is proposed and its main building blocks and layers are thoroughly described. Aiming to offer a broad perspective on the business potential of such a platform, the stakeholder ecosystem is also analyzed. Finally, two use cases in the context of smart cities and mobile health are presented, aimed at showing how the proposed PaaS enables the development of respective IoT applications.

## 1. Introduction

The rapid proliferation of the Internet of Things (IoT), comprising connected sensors and devices, provides opportunities to develop intelligent applications, transforming data into business knowledge and societal information for a broad set of verticals, from smart grid and autonomous driving to industrial automation and sports entertainment. The IoT growth is further fueled by multi-access edge computing (MEC) advances and fifth generation (5G) mobile communications, providing architectures, platforms and tools, for IoT and cloud computing integration in the network softwarization era. However, current IoT devices’ deployment not only includes resource-constrained sensors and actuators, but it is also extended to embrace heterogeneous smart devices. Thus, a novel ecosystem is formulated, where hybrid cloud resources may collaborate within a single management context setting, referred to as resource continuum [1], cloud to thing continuum [2], or fog-to-cloud [3], among others. A hybrid cloud combines two or more cloud environments, i.e., a public cloud and a private cloud enabling the sharing of data and applications among them. Hybrid clouds provide increased flexibility for organizations to scale computing resources, reduce capital expenditures while handling peaks in demand and facilitate the allocation of local resources for more sensitive data or applications. In this ecosystem, incipient distributed and federated combinations of infrastructure located close to the edge reduce latency, minimize network load and optimize services execution, through data and computational offloading [4].

### 1.1. Background

In order to cope with the requirements of data-intensive IoT applications on fog-to-cloud fabric, modern cloud platforms, such as Amazon Web Services [5], Google Cloud IoT Edge [6] and IBM Watson IoT Cloud [7] facilitate their development, deployment and provisioning, offering convenient hosting options and delivery models, e.g., Infrastructure-as-a-Service (IaaS) and Platform-as-a-Service (PaaS). Moreover, they offer a rich variety of services to support cloud-native applications, as well as management and orchestration of computing, networking, and storage infrastructure on behalf of user workloads. In addition to these offerings, modern cloud platforms provide solutions for IoT data processing and analytics of complex flows of events and data, enabling efficient ingestion and aggregation of data streams generated by the IoT devices located at the edge. Thus, the current trend focuses on hybrid data lakes and fog-to-cloud fabrics facilitating the processing of data in dynamically created and updated pipelines, located close to where the data are, with no need to forward data for processing at far cloud premises. However, such a smart fog and cloud integration is not free; instead, novel management strategies must be designed for successful resource orchestration, intended to facilitate the dynamic allocation of cloud services wherever the proper resources might be. Indeed, developing an accurate and service agnostic resource management into an integrated edge/fog/cloud fabric would accommodate the deployment of new services, innovative artificial intelligent (AI)-based decision-making, proper cloud-fog offloading strategies and novel business models, considering the full set of resources from the very edge up to the cloud. To that end, innovative resource discovery, monitoring, and categorization strategies must be designed to overcome the challenges related to the deployment of edge devices, i.e., mobility and some control policies in use to “handle” them, e.g., control battery consumption, usage time, and others.

### 1.2. Challenges

**Figure 1:**
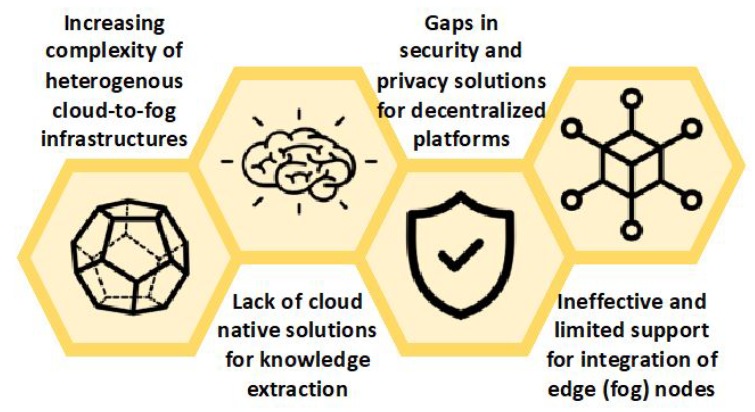
Current challenges in hybrid clouds for data-intensive IoT applications.

While the aforementioned ecosystem fosters innovation in IoT-related markets and industries, in many cases, the full potential of IoT is hindered by several challenges that have to be properly addressed. Figure 1 presents the main challenges of enabling data-intensive IoT applications in hybrid clouds.

First, there exists increased complexity in managing heterogeneous fog-to-cloud infrastructure. More specifically, there are numerous cloud architecture paradigms, ranging from hyperscalers to micro data centers close to the edge, satisfying diverse user requirements, with each one holding its unique characteristics. However, in spite of hybrid cloud being an extremely important enterprise paradigm, there are still important open issues to be addressed, related to intermittent connectivity, transparent support, policy enforcement management, management of resources under strict service level agreement (SLA) conditions, as well as privacy and anonymization issues related to data and workload portability across the hybrid cloud.

Then, the lack of cloud native solutions for knowledge extraction should be considered. Forthcoming IoT applications will demand efficient knowledge extraction from data located in different areas of the decentralized hybrid clouds and within data lakes in the form of unstructured data. Still, the simplicity of a central data lake model combined with reliable and efficient distributed cloud-native computation and privacy-aware data access to train models is missing. In addition, such model training must be performed in a distributed and federated manner, efficiently extracting the knowledge, exploiting the serverless computing principles [8].

In addition, several gaps in security and privacy solutions for decentralized platforms threaten their reliable operation. As the development and use of Internet technologies increase, security threats and violations are becoming commonplace and more challenging to manage. To cope with the gap of security issues, it is essential to exploit dataspaces [9]. In this way, different cloud platforms can be connected through secure exchange and trusted data sharing with novel encryption algorithms. Moreover, the use of sandbox environments for data validation should be promoted, overcoming possible problems encountered in conventional data integration systems.

Finally, currently, the support of integrating edge and fog nodes in hybrid clouds is ineffective and limited. In the majority of cases, cloud-to-edge dynamic and transparent data processing is not yet a reality, and this is a major drawback for the further adoption and uptake of IoT applications. The solutions that are proposed until now cannot provide immutability and are prone to errors. Furthermore, these solutions are lacking automated decision capabilities with respect to determining when data or computations must be offloaded in a hybrid, fog-to-cloud environment. This shortcoming demands for: (1) tailored AI algorithms to apply automated fog-to-cloud offloading/orchestration; (2) distributed identity management and accounting solution facilitating resource sharing to enable collaborative offload of computational tasks to the edge nodes.

### 1.3. Contributions

Taking into consideration the challenges in the field of MEC hybrid clouds for IoT applications, in this paper, an architecture for a PaaS is proposed. This novel PaaS capitalizes on Cloud Native Computing Foundation (CNCF) [10] and Apache Software Foundation [11] open source projects. In addition, by providing a detailed overview of the architecture and its building blocks, its benefits for simplifying the development and dynamic orchestration of cloud native, data-intensive and intelligence driven IoT applications are clearly given. More specifically, the contributions of this work are as follows:

- A thorough literature review on the main relevant areas is given, including anomaly detection, monitoring frameworks, identity management and fog node discovery among others.
- The presentation of an innovative architecture for supporting the dynamic provisioning and management of decentralized, cloud-native, MEC-based, data-intensive IoT applications across hybrid clouds under data sovereignty, security and trust constraints, is presented.
- Several business aspects are discussed, identifying the main stakeholders, aiming to maximize the impact of the proposed PaaS architecture, as well as its uptake by interested parties.
- Use cases of IoT scenarios in the context of public sector and mobile health (mHealth) are presented, depicting the benefits of adopting the proposed MEC-related PaaS architecture to support data-intensive IoT applications.

### 1.4. Outline

This paper is organized as follows. In Section 2, a literature review is given, while Section 3 presents the proposed hybrid cloud architecture and its building blocks. Section 4 lists the stakeholder ecosystem. Next, Section 5 presents two potential use cases for deploying the proposed PaaS. Finally, Section 6 includes the conclusions of this work.

## 2. State-of-the-Art and Key Issues

Here, relevant research areas for developing hybrid cloud solutions are discussed. Then, for each area, current shortcomings are given, highlighting the need of designing the proposed PaaS.

### 2.1. Anomaly Detection, Root-Cause Analysis and Mitigation

Monitoring systems of cloud infrastructures are often designed as: (1) computation-based focusing among others on CPU usage, memory usage and I/O operations and (2) network-based examining the performance of throughput, latency, jitter, packet loss, etc. This process is crucial to assess cloud resources or applications continuously in terms of performance, reliability, security, power usage, ability to meet SLA requirements, etc. [12]. In IoT, billions of devices enable services in environments such as smart city, smart traffic, smart home, smart healthcare and are often hosted in various deployments as it is the case of decentralized cloud, fog, edge, and IoT, providing the services of multiple providers. Characteristics of decentralized cloud components include the security and performance of cloud-based services. Decentralized clouds offer compartmentalization of risks, whereas centralized clouds are threatened by zero-day attacks [13]. To enable secure services, anomaly detection, diagnosis, and mitigation is needed to ensure Quality of Service (QoS) and reliability. In a multi-dimensional view of anomalies in decentralized infrastructures, two major anomaly categories are performance anomalies and security anomalies, resulting in QoS degradation, SLA violation, interruption, denial of service (DoS) and nasty port scanning.

Unfortunately, the existing solutions have several shortcomings mainly revolving around two major areas. First, current anomaly detection does not address the detection of performance and security anomalies e.g., hyperscale and multilayered attacks, such as botnet, Distributed Denial of Service (DDoS) in the cloud-native era, required to ensure QoS and reliability at scale from resource-constraints devices to data centers. In order to support automated detection, relevant mechanisms employ agent feedback-based learning algorithms. Secondly, root-cause analysis and mitigation of such attacks at an early stage without service interruption in decentralized settings are needed. Possible solutions should adopt the kubeflow of the Kubernetes framework to enhance performance and security.

### 2.2. Monitoring Frameworks in the Cloud-Native Era

Infrastructure and application monitoring are essential in federated cloud environments, given the highly distributed and dynamic nature of deployment and orchestration of clusters and pods, as well as the heterogeneity of hybrid, cloud-to-fog technologies and fast allocation of resources, under the serverless paradigm. These unique characteristics bring specific challenges currently not addressed by existing monitoring solutions. General-purpose monitoring tools, such as Nagios [14] or Zabbix [15], are used by system administrators for fixed or slowly changing distributed infrastructures and respective services. Collectd [16] and Netdata [17] are network monitoring and metric collection tools, lacking the providing of a holistic approach covering the cloud native approach. Snap [18] is a highly-extensible open-source telemetry framework designed for cloud-scale monitoring of the complete software and hardware stack. cAdvisor [19] is a utility that runs in a Docker container and collects useful information about the services on running containers. However, these solutions do not achieve the requirements stemming from the applications and infrastructures envisioned in the cloud native landscape, by being intrusive and heavy-handed for short-lived, lightweight network function instances, not following the fast pace of management changes enforced by continuous dynamic scheduling, provisioning and auto-scaling of applications and not covering the requirements of all the involved emerging technologies, including deployments in both a hypervisor-based and containerized manner, as well as monitoring data collection from federated clouds.

Considering the frequent instantiation, deletion, migration and scaling of applications in a hybrid cloud-to-edge environment, there is a need to design a monitoring system for dealing with the dynamic and distributed nature of microservices deployed in such heterogeneous networks, capable of collecting, aggregating and distributing monitoring metrics between various components. A suitable monitoring framework must consider, as a starting point, the CNCF projects related to monitoring and provide a monitoring toolbox that can be tailored according to specific needs of the developers, application providers and infrastructure owners. From the current CNCF constellation, various projects can be identified, such as Prometheus, Cortex, fluentd, cAdvisor and OpenMetrics.

### 2.3. Identity Management and Accountability

Currently, identity management is mostly based on centralized solutions, such as corporate directory services, domain name registries, or certificate authorities. However, these approaches are fragmented and siloed between various service providers, limiting the adoption of a holistic view and thus delivering poor user experience, due to repetitive registrations and logins. The upcoming reliance on billions of IoT devices makes it untenable to have all those devices controlled by a centralized identity provider, since a breach of this provider would be disastrous not only for revealing personal data and misallocation of virtual resources but also for attacking the physical infrastructure including the IoT devices. The emergence of distributed ledger technology (DLT) offers a promising solution, providing the opportunity for fully decentralized identity management [20]. This technology pushes ownership of identity away from centralized services to the edge to individuals so that the identities themselves are in control [21]. Bitid [22] is an open protocol which allows simple and secure user login to cloud/web services with authentication based on the public key and blockchain-based network. OpenID [23] and NameID [24] are open protocols that allow a user to authenticate to multiple services, providing one unique identity to the user from some trusted identity provider. Finally, uPort [25] is a platform for end-users to establish a digital identity that can be used as user identity across multiple services, giving the users full control of sensitive data, and their digital assets, securely and selectively disclosing their data to counterparts, accessing digital service. Finally, the Blue Horizon IoT Blockchain initiative is targeting a fully symmetric distributed setting, being used within the IBM cloud platform [26].

These solutions have two shortcomings that necessitate novel approaches in hybrid cloud environments. First, they do not address issues of multi-tenancy, encryption, privacy-preservation and scalability in the emerging cloud-native era. Then, they provide self-sovereign identity, mainly for authentication and authorization purposes. However, a complete solution must address accountability, and requirements from the standpoint of the protected services, e.g., user authentication and accountability, considering computation offload and data portability from resource-constrained devices to hybrid clouds.

### 2.4. Service Level Agreement Management

The Open Grid Forum (OGF) describes the WS-Agreement standard, for SLA creation, as a protocol for “establishing agreement(s) between two parties, such as between a service provider and a consumer, using an extensible markup language (XML) for specifying the nature of the agreement, and agreement templates to facilitate discovery of compatible agreement parties” [27]. This standard has been considered during the implementation of several SLA tools, to different levels of success, mostly from an infrastructure-oriented perspective. This is the case of most common tools used in cluster management, such as the ones integrated by Kubernetes [28] or Openstack [29]. While these tools are widely used, they are also very restrictive, forcing the users to express their constraints at a low, infrastructural level. SLAs need to simultaneously describe high level requirements from different perspectives, an option only provided by SLA-oriented tools. These tools are commonly designed as part of particular deployments, to which they are tightly dependent. This is the case of WSAG4J [30], a Java framework for SLA management and SLA@SOI [31], a service-oriented infrastructure. These solutions directly implement the WS-Agreement standard, but have extensive computation and storage requirements, making them unsuitable for fog/edge deployments.

In hybrid clouds, the automatic SLA management diverges from restrictive SLAs from a system perspective that are integrated by most cluster management tools. This enables tailored negotiation and more complex SLAs for provider and user requirements. This is particularly relevant for fog and cloud native deployments, working on flexible architectures scaling up and down resources that have limited computing power most of the time.

### 2.5. Anonymization and Encryption

Inherent data security and privacy areas in cloud-to-edge fabric include: (1) lightweight and fine-grained data encryption and sharing methods, (2) distributed access control under resource constraints and (3) preservation of data privacy among heterogeneous environments [32]. Thus, hybrid cloud environments have emerged to guarantee data privacy and integrity before and after the offloading of data among nodes in cross-layers. In this context, anonymization and encryption facilitate data collection, processing, storage and dissemination either on-premises or at node level in hybrid clouds. The uncovering of private data results in violations of legal framework, most notably the General Data Protection Regulation (GDPR). In the cloud-to-edge fabric, major concerns consist of the collection of sensitive data from the end-user devices and their aggregation for analytics, the protection of end-user identity during authentication and management, as well as end-user location privacy [33].

Novel anonymization and encryption approaches should address two primary problems in hybrid clouds. Firstly, anonymization enforces the fine-grained techniques (e.g., attribute-based anonymization) to obscure the sensitive data before it is stored and later provides accessibility, benefiting developers in the cloud-native era, while being compliant with legal and regulatory frameworks. Secondly, data encryption enforces the lightweight techniques as e.g., homomorphic encryption, probabilistic public key encryption to encode the sensitive data, mitigating data breaches. It should be noted that anonymization and encryption must be performed in an automated or on-demand manner for data privacy in hybrid clouds.

### 2.6. Fog Node Discovery

In order to fully leverage the spare computational capabilities within fog nodes (FNs), a suitable mechanism, ensuring a timely discovery of those nodes needs to be implemented. Relevant literature includes a few contributions on resource discovery in the fog/edge computing. In FogOS [34], two discovery approaches are envisioned: a proactive approach where the FogOS-administered network is notified whenever a device willing to join the system is present and a reactive approach where edge devices are queried on demand for their availability. In the OpenFog Reference Architecture (OFRA) [35], a new FN advertises its presence via broadcasts. Nonetheless, these approaches do not provide a real-world implementation of the discovery mechanism. In Foglets [36], the discovery server is a partitioned name server with a periodically-updated list of FNs residing at the different levels of the fog-cloud hierarchy, while, in Edge-as-a-Service (EaaS) [37], a master node listens on a specific port for discovery requests from edge nodes willing to contribute to the system. Regarding resource discovery from the client’s perspective, cloudlet discovery is performed using DNS-Service Discovery (DNS-SD, RFC 6763 and Multicast DNS (RFC 6762) [38].

In data-intensive IoT applications relying on the deployment of numerous fog nodes, it is necessary to devise a discovery solution that can be easily implemented and integrated, providing location- and context-awareness. Such a solution must consider the dynamic nature of edge devices and their geo-distribution, as well as the fact that they do not necessarily belong to a single networking domain. Additionally, given the heterogeneity of edge networking technologies, it needs to abstract the underlying technology. Finally, if a client-side discovery mechanism is considered, client-related constraints, such as energy limitations and latency constraints, need to be taken into account.

### 2.7. Hybrid Cloud Orchestration

Hybrid cloud orchestration through resource federation from multiple cloud providers is as old as cloud computing itself. More specifically, the concept of cloud federation was pioneered by the Reservoir FP7 project over a decade ago [39]. Subsequent research proposed ways to form federation at the cloud provider level by means of a layered service model [40] and proposing a cross-cloud federation manager with discovery and authentication [41]. Other works discuss resource allocation in cloud federations [42] and architectures for federated clouds [43]. Several approaches for application deployment in hybrid clouds with various optimization objectives have also been proposed [44]. Recently, Kubernetes has become the de-facto orchestration and scheduling platform for containerized applications, offering portability of applications across diverse cloud infrastructures. Related activities such as Crossplane [45] focus on automating application deployment on infrastructure from multiple providers. Mesosphere DC/OS 1.11 [46] includes a unified control plane for multi/edge cloud operations, and multi-layer security. These solutions require one to setup the underlying clusters first and then join the clusters manually to the management control plane. They assume that underlying clusters and devices are mostly static and, although clusters can be added or removed from the federation, this can only be done manually by the system administrator.

Novel paradigms of hybrid cloud orchestration should target the transformation of the federation lifecycle management from a manual procedure into an automatic process performed in an optimal manner. To maximize the gains of federation lifecycle management, extensions to Kubernetes and its federation Application Programming Interfaces (APIs) [47] must be provided by the orchestration frameworks.

### 2.8. Serverless Hybrid Data Lake

Serverless data lake architectures are widely adopted by the industry [48]. Typically, a serverless data lake is architected as a centralized cloud-based solution. The data are organized using catalogs that hold meta-information about the data objects. Events related to the data objects appearance or disappearance trigger serverless computations that can be stacked together as data analytic pipelines. The pipelines can be very complex. In addition, the workloads can be irregular. This calls for serverless workflow management systems. Initially, serverless computing was oriented towards short term stateless computations. To overcome this limitation, specialized serverless orchestration systems have been introduced, such as AWS Step Functions [49], Azure Durable Functions [50], and Apache OpenWhisk Composer [51] and compared [52]. Nonetheless, current solutions suffer from various limitations. In particular, the current serverless orchestration systems are intrinsically centralized, they are not portable across clouds, their state is not shareable among different flows and they are not built to mix and match multiple paradigms.

Thus, a radical departure from current approaches is needed. More specifically, the development of a cloud native State-as-a-Service (SaaS) mechanism decouples the state from the serverless execution in a transparent manner. In addition, technologies, such as Knative, are able to provide this service cost-efficiently. In essence, a serverless computation can be implemented as a state machine, resuming from the last consistent state responding to events in a distributed data lake. Then, leveraging K8s’ federation will lead to a truly distributed serverless flow orchestration system, spanning multiple K8s clusters. Portability can be achieved by using K8s as a standard container orchestration mechanism, while cloud native service mesh technologies, such as Istio [53] mix and match different computing paradigms within the same serverless state machine.

An overview of the current state-of-the-art and the respective key issues in each research area are included in Table 1.

## 3. Architecture of a Novel PaaS Solution

Here, a relevant architecture aimed at addressing the current shortcoming of hybrid cloud environments is presented. More specifically, first the general approach for building the innovative PaaS is given and then each layer is discussed in detail.

### 3.1. Concept and Approach

**Figure 2:**
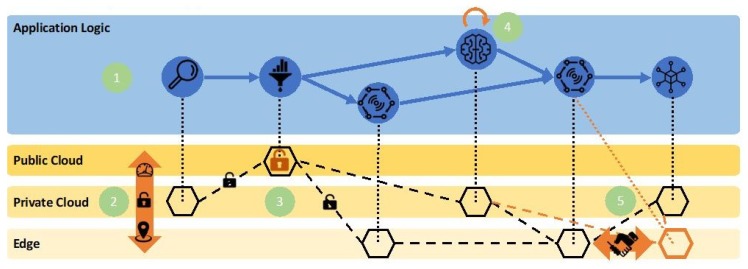
The five tasks of the Platform as a Service (PaaS) concept.

The main target of the proposed PaaS is to provide a platform, facilitating the deployment and management of IoT applications and data pipeline orchestration across the edge-cloud continuum with security and privacy guarantees. Figure 2 depicts the five tasks that are performed in this PaaS across the different cloud domains. More specifically, the platform aims at enabling: (1) the definition of cloud native IoT application logic at an abstract layer decoupled from deployment, security and privacy constraints; (2) the governance, through automated intelligent decisions, of the edge-cloud continuum, taking into account localization, performance and other security constraints, such as the dynamic binding and continuous optimization of the application logic to specific resources; (3) the creation of secure data sandboxes when data cross the barriers of different domains to leverage third party resources, e.g., public clouds or shared edges; (4) the automated management of complex tasks in the machine learning edge-cloud flow, ensuring an optimized machine learning lifecycle, including model training, model configuration and model execution loop; and (5) infrastructure collaborative sharing and offloading, ensuring immutability of data processing.

The overall approach for building this PaaS revolves around three pillars. The first pillar deals with the realization of a cloud-to-fog fabric for the dynamic resource federation across the hybrid cloud and the edge, including nodes that, while providing constrained connectivity, networking, computing and storage capabilities, are close to data generation and actuation. The second pillar provides security and privacy guarantees to address end-users and business concerns, further stimulating the utilization of hybrid clouds. Then, the third pillar is focused on the intelligent orchestration of data pipelines and runtime execution environment, adopting and integrating the serverless computing paradigm.

**Figure 3:**
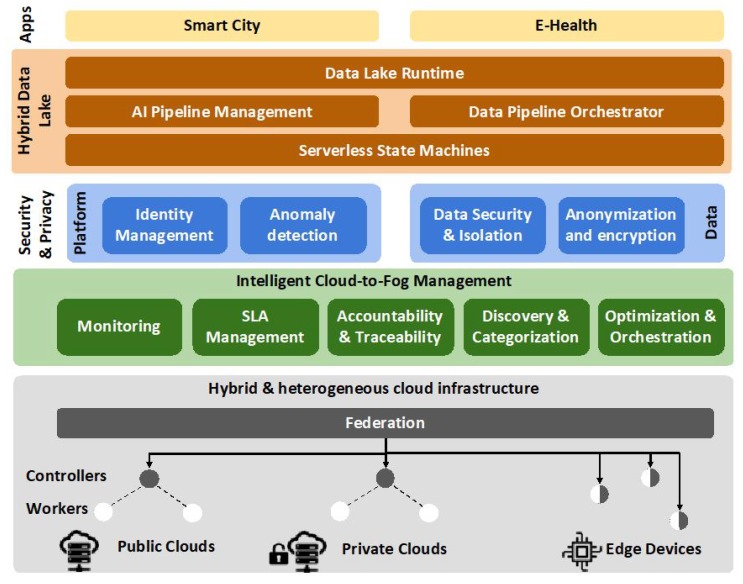
Architecture diagram and building blocks of the proposed PaaS solution.

A high-level architecture and its building blocks in the hybrid, cloud-to-fog environment is depicted in Figure 3, where the three pillars are translated into three main layers. The first layer on top of the cloud infrastructure is the Intelligent Cloud-to-fog Management layer, dealing with the management, orchestration and optimization of resources. The Security and Privacy layer handles security and privacy in two different levels; platform and data processing. The third layer is named Hybrid Data Lake and aims to orchestrate and enable data pipelines to support different data driven applications, being served by the Data Lake API. On the infrastructure layer, each cloud consists of different Kubernetes worker nodes controlled by a control (Kubernetes master) node. On top of the control nodes, a Kubernetes federation layer resides, defining and managing clusters across the clouds in the public, private and fog domains.

### 3.2. Intelligent Cloud-to-Fog Management

The Intelligent Cloud-to-Fog Management layer consists of five main building blocks described next, and is responsible for the management and orchestration within the cloud infrastructure.

The Monitoring toolbox is responsible for: (1) retrieving performance data and security logs from running services, applications and software components running on different nodes, (2) implementing a data modelling/indexing schema for adapting and pre-processing of the collected data in a homogeneous and standardized manner, (3) storing data in a persistent database, accessible by other platform components, for intelligent resource management, data portability decisions, etc. To that end, the toolbox relies on CNCF tools, including monitoring frameworks (Prometheus [54], Cortex [55]), monitoring data collection (cAdvisor), logging capabilities (fluentd [56]) and standardized approaches for data modelling (OpenMetrics [57]).

The SLA Manager ensures an agreed level of service for infrastructure, i.e., what resources are provided and data, i.e., what data are provided and how they are managed. In particular, the management layer extends tools, such as SLALite tool [58], adapted from the infrastructure perspective (non cloud-native approach) to a data-centric one (cloud-native approach). This adaptation is done by extending: (1) the existing ontology of requirements to represent data owners requirements that is, what are the basis of the services, offered by a data provider to the cloud deployment; (2) the SLA models to represent not only the interaction between the infrastructure provider and the customer, but also between infrastructure and data providers; and (3) the automatic creation of SLAs, so that, based on the requirements of each stakeholder, the system proposes SLAs meeting these requirements.

The Accountability and Traceability component relies on distributed ledger technologies, offering immutability and traceability of data and computation offloading across the fog continuum and providing collaboration among different stakeholders by sharing edge resources. Leveraging existing platforms, such as Ethereum and Hyperledger, the Accountability and Traceability component offers consensus-based accounting, based on smart contracts without a supervising authority, thus providing a novel approach, going beyond current solutions. Computational offloading is accounted, based on technical and non-technical metrics, e.g., processing power, network delay, pricing and privacy-preservation restrictions in collaboration with the SLA Manager.

The Resource Discovery and Categorization component encompasses features related to the discovery, categorization and abstraction of the edge resources. The resource discovery module provides mechanisms, allowing such resources to advertise their presence in a seamless and timely manner. The discovery mechanism handles the heterogeneity, mobility, volatility and intermittent connectivity characteristics, which are inherent to edge scenarios. Then, once edge resources are discovered, the categorization module extracts their underlying characteristics. Such characteristics may include their capabilities in terms of CPU cycles, storage, available network interfaces, etc. Finally, physical resources are grouped into logical clusters, using the abstraction module. Such clusters should be set according to policies responsible for optimally managing the inherent characteristics of edge devices.

The Resource Optimization and Orchestration component provides the orchestration mechanisms for hybrid cloud operations in the fog-to-cloud fabric. This fabric takes a starting point in the Kubernetes federation API v2, providing basic operations for joining cluster into federations and deploying workloads. Notably missing from these technologies, but key for the operation of the proposed architecture, are context-aware optimization and lifecycle management mechanisms to manage heterogeneity, partial connectivity, and application-aware management. The proposed architecture provides these key aspects in the fog-to-cloud fabric.

### 3.3. Security and Privacy

The Security and Privacy layer takes care of security and privacy considerations, and is handling both the platform level and the data level. The layer consists of four components, as described below.

On the platform level, the Identity Management component provides authentication and authorization to platform users, based on distributed ledger and smart contract technologies.

Then, Anomaly Detection deals with the detection, diagnosis, and mitigation of anomalies (from IoT-related to hyperscale and multilayered attacks) that cause security violations across the host, Virtual Machine (VM), containers, applications and API services. This is essential to ensure QoS and reliability across the fog-to-cloud fabric. This component leverages on interactive learning agents deployed at each layer and node, to continuously feed the data to the monitoring component to detect and mitigate anomalies. These self-healing agents recommend mitigation of anomalies enabling automated corrective actions. The main novelty of the proposed architecture’s Anomaly Detection is its ability to automate the detection of anomalies across layers, e.g., serverless functions and applications in cloud-native environments. The mechanism aims at employing interactive learning algorithms, capitalizing on kubeflow.

On the data flow side of security and privacy layer, the Anonymization and Encryption component provides privacy functionalities to protect sensitive or personal data from unintended disclosure, before and after offloading from one node to another. Specifically, it provides anonymization of sensitive data before it is stored, aligning with legal and regulatory frameworks. This process securely and irreversibly anonymizes data, e.g., using attribute-centric anonymization, without deleting records that de-identifies the data subjects, whilst maintaining data consistency in the linked system. Precisely, data encryption, e.g., using homomorphic encryption mitigates inadvertent disclosure and damage from data breaches. This service can be used in an automated or on-demand manner, to ensure privacy among data of cross-layers in hybrid clouds.

Additionally, the Data Security and Isolation component provide data privacy and security infrastructure, as a wrapper layer around data processing, decoupling the data processing business logic from the logistics concerns of data access. In greater detail, it enables the application developer to focus on the data processing logic by addressing data access control, privacy concerns and the required governance and compliance. It bundles anonymization and encryption within the application logic in a secure and decoupled manner from the application logic. The security and isolation align with pipeline orchestration specification and data lake runtime guarantees, including processing location and context.

### 3.4. Hybrid Data Lake Management

The Hybrid Data Lake layer enables the composition of serverless, micro-services and services together into data-intensive and intelligence-driven data lake pipelines with optimized performance, cost and subject to policy compliance, security guarantees, and SLA enforcement. This layer is built around two concepts: (1) policy driven serverless data processing and (2) distributed execution pipelines making use of specialized runtimes.

First, the Data Lake Runtime provides the foundations of the cloud native hybrid data lake. It is composed of cloud-native middleware components and creates a managed environment for running data processing code in a compliant and secure way. Such data driven pods can be orchestrated to run on the cloud or edge, considering the specification in the data catalog, the function catalog, and the runtime environment, be it cloud or edge, and its specific restrictions.

Furthermore, the Serverless State Machine supports the data pipelines across the serverless components in a unified manner. As a starting point, solutions such as K8s Knative serverless framework will be considered. This service provides the crucial “glue” to manage the data lake pipeline in an efficient and dependable manner in the hybrid cloud. The state machine will seamlessly integrate with the Data Pipeline Orchestrator, creating a first of a kind integrated distributed data pipelines orchestration system, allowing developers to benefit from different programming paradigms.

Then, the Data Pipeline Orchestrator (DPO) automatically orchestrates dynamic data pipelines over cloud and edges, using context information, i.e., system resources, data entities’ metadata and QoS requirements. The DPO can be built around different open source technologies, such as FogFlow [59], Apache OpenWhisk Composer [60] and Apache AirFlow [61], implementing new functionalities for automated and optimized IoT applications orchestration and data-lake specific optimizations, including data privacy enforcement. More importantly, the DPO should enable serverless edge computing, so developers can define and submit fog functions that will then be triggered, deployed and run, providing automated and optimized orchestration with high scalability and reliability. Fog functions will be implemented following a data-centric programming model and a development toolchain for developers and system integrators, allowing the development of application at a low cost and with a fast time-to-market.

Finally, the AI Pipeline Manager provides configurable and serverless AI tasks, as building blocks for data pipelines. This component dynamically manages the lifecycle of AI tasks in the data pipeline by creating models based on the specific task to be run and the evolution of data used in the pipeline, supporting the continuous training of AI models. DPO enforces QoS for data pipelines, including AI tasks through the adaptable composition of the flows, optimizing the flows according to their dependencies, as well as the dependencies on the data lake structure. To support the creation of AI tasks and the adaptable composition of pipelines, advanced knowledge extraction and real-time situation awareness are required, towards developing tools with unique capabilities for the implementation, deployment and management of IoT applications.

## 4. Business Aspects

An important step towards ensuring a wide adoption of relevant hybrid cloud architectures is the identification of the stakeholder ecosystem. In what follows, these stakeholders are listed and a discussion is provided for each one.

The first category includes industrial stakeholders, as the platform facilitates the development of IoT applications and smart services, based on highly distributed data-intensive and widely connected fog/edge nodes in hybrid clouds. As a result, industrial stakeholders developing applications for the private and public sectors, e.g., e-Health, smart Cities, intelligent transportation, will find such PaaS architectures highly attractive.

Then, there are the IaaS hybrid-cloud providers that are offered interesting opportunities to foster new business models, through hybrid cloud deployments. Nowadays, cloud infrastructure providers offer networking resources, but still these resources are underutilized and mismanaged and might not satisfy the service requirements in fog/edge computing scenarios, involving edge devices with limited capabilities. Hybrid clouds, as considered within the proposed platform, allow local infrastructure providers to exploit the availability of public cloud resources and ensure satisfactory capacity for highly demanding applications in IoT scenarios.

Another important stakeholder category includes small and medium-sized enterprises (SMEs) and developers. An advanced architecture for data-intensive IoT environments is of significance to SMEs working on addressing challenges in developing cloud-native, data-intensive applications and integrating novel concepts, such as serverless computing and AI-enhanced orchestration. Moreover, developers can exploit the fast development cycle, the monitoring tools, the data access control, as well as the various programming environments, supported through the proposed architecture.

As hybrid cloud architectures should be built around open source tools, open source communities are highly important for the success of such PaaS solutions. The proposed PaaS architecture capitalizes on tools from existing communities, e.g., CNCF, Apache projects, FIWARE, and others. Thus, it is important to develop such platforms in alignment with on-going open source activities, being of relevance to the open source communities.

Moreover, policymakers play a pivotal role in influencing ICT investment strategies and redirect academia and industry towards adopting new technology paradigms. Hybrid clouds should comply with legislation and other policy requirements to increase the support, towards future cloud-native data-intensive applications that will span across several cloud domains.

In order to guarantee the adoption in different markets, standardization and industrial groups should be attracted to novel hybrid cloud approaches. Currently, there are several standardization and industrial initiatives on areas that are highly relevant for the cloud and IoT domains, such as the FIWARE foundation, Future Internet and 5G public private partnerships (PPPs), NetWorld2020, the The Alliance for the Internet of Things Innovation (AIOTI) and others.

Finally, hybrid cloud deployments should accelerate the transformation towards smart cities. This category comprises a wide group of stakeholders including citizens, students, and public authorities that could benefit from cloud-native, data-intensive applications.

## 5. Use Cases

The proposed PaaS platform could be applied to several IoT scenarios, where efficient management of fog-to-cloud infrastructure is important. In this section, we present two potential use cases, i.e., the smart city maintenance scenario and the personalized medicine scenario and we discuss the expected benefits and costs derived by the adoption of the proposed PaaS platform. Table 2 summarizes the expected benefits, costs in terms of relevant infrastructure applied for each scenario and relevant stakeholders.

### 5.1. Leveraging on Geo-Distributed Urban Data Assets for Smart City Maintenance

The maintenance of cities infrastructure is a fundamental activity to ensure that city services work properly, requiring continuous monitoring and assessment of the infrastructure status. Nowadays, monitoring is mostly done by public officers, and, in some cases, by citizens through standards, such as Open311 that enable citizens to report issues affecting their neighborhood. Nonetheless, this feedback needs validation by city officers, before planning any corrective action. In several scenarios covered by Open311, automatic techniques would be applicable to detect and confirm, thus reducing the efforts to monitor infrastructure conditions and validate reported issues. One of the most solicited public infrastructures are roads, where proper maintenance is fundamental to reduce road accidents, guarantee traffic flow and contain road rehabilitation costs.

To improve the maintenance procedures, several municipalities are looking for innovative solutions for road infrastructure monitoring to detect potholes, deteriorated road signs and other similar issues. Recently, Wolfsburg Municipality demanded Wobcom, the public company managing the city IT infrastructure to explore innovative solutions, such as equipping public busses with high resolution cameras and sensors to perform automated and distributed road monitoring across the city [62]. To combine the detection and human validation of detected issues, it is important that detection occurs in real time over the buses, so that the bus driver can easily confirm the detected issues. This process requires that AI detection algorithms are on board the bus, guaranteeing their operation even when connectivity is not available. Moreover, models underlying the AI algorithms need to be constantly updated to improve their efficiency and the spectrum of detected issues. Contrary to detection, models are more efficiently computed based on a large set of training data, thus their computation is more suitable in centralized infrastructures. This demands for a way to orchestrate in a decentralized infrastructure and not always online, algorithms and data. Thus, it is necessary to implement a fog architecture, including nodes located on busses and a central computation service in a data center, integrated within the smart city platform.

The challenges identified in this use case are: (1) the collection and fusion of geo-dispersed multi-modal data streams (e.g., camera images, event reporting, etc.); (2) the support of human sensors enabling citizens to report events in a traceable manner; (3) the intelligent distribution of AI algorithms from the edge up to the core infrastructure supporting real-time issue detection; (4) the delivery of intelligent services supporting smart city maintenance to urban stakeholders. In order to address these challenges, the proposed PaaS solution can (1) optimize the performance and precision of the AI algorithms in the fog-edge continuum, enabling the delivery of real-time smart city maintenance services to city stakeholders; (2) trigger automated actions ensuring that issues are mitigated or solved in the short time possible; (3) test the serverless approach, enabling the flexible on demand deployment of data processing tasks upon the detection of events.

### 5.2. Connecting Health Data Lakes in A Trusted Environment Supporting Personalized Medicine

The rise of mHealth applications, capable of continuously monitoring health conditions of patients and the adoption of Electronic Health Records (EHR) from healthcare providers offers a wealth of medical data to build personalized medical services and allow early prediction and prevention of diseases [63]. In particular, the use of remote monitoring in patients with chronic diseases could significantly improve healthcare costs efficiency and reduce the need for doctor’s visits and hospitalization [64]. However, the potential of IoT and cloud technologies in the healthcare sector is still in its early stages due to: (1) lack of integration among applications owned by different healthcare stakeholders, such as hospitals, insurance companies, telecare providers, etc. and (2) low adoption and performance of AI techniques to support decision-making at micro-level (patient-centric). As a result, the digital solutions in the health sector remain largely fragmented, leading to data silos and suboptimality in the delivery of intelligent healthcare services.

This use case requires the collaboration among IT healthcare providers, offering IT products based on cloud computing and IoT technologies. Such technologies consist of three layers: (1) personal area layer, equipped with medical devices, wearables, remote sensors and wireless patches for patient status monitoring and data transmission to a gateway, (2) fog layer, consisting of edge cloud infrastructure, providing a first level of support, guidance and recommendation to the patient without the need for communicating with the centralized cloud of the hospital and (3) core cloud layer, corresponding to central cloud infrastructure hosting services/applications of the hospital, e.g., decision support tools for doctors, HER.

The challenges towards the realization of hybrid cloud solutions for mHealth applications are: (1) the connection of dispersed health data lakes through a secure interconnection framework for infrastructure of different administrative domains; (2) the efficient management of a federated platform hosting healthcare services, guaranteeing SLA compliance; and (3) the authentication of different stakeholders in the healthcare platform and the enablement of new business models relying on the exploitation of health data and services across federated platforms. In order to tackle these issues, the proposed PaaS solution can deliver optimized AI-based services relying on a decentralized architecture for the deployment of AI algorithms. In this way, data can be shared among different stakeholders in a secure and privacy-aware manner, validating the performance of intelligence tools on top of decentralized data sets.

## 6. Conclusions

In this work, hybrid cloud deployments for supporting data-intensive, 5G-enabled IoT applications were investigated. More specifically, an overview of the state-of-the-art in hybrid clouds and relevant areas, including anomaly detection, anonymization and serverless data lakes among others, was provided. Moreover, several key issues were identified towards the realization of efficient deployments for satisfying the requirements of challenging MEC-related IoT applications not only in terms of the data volume, but also service level agreements, as well as security and privacy constraints. Then, a decentralized hybrid cloud architecture was presented in detail and its main building blocks were analyzed. The business impact of such a Platform-as-a-Service was discussed, and relevant stakeholders were highlighted. Finally, use cases in the context of smart cities and mobile health were presented and the role of the proposed platform in addressing their requirements was given.

## Figures and Tables

**Table 1:** Relevant research areas for hybrid clouds with their current advances and key issues.

Research Area	State-of-the-Art	Key Issues
**Anomaly Detection**	Cloud resource assessment, application performance monitoring, risk compartmentalization	Hyperscale and multilayered attacks, automated detection, early stage root cause analysis and mitigation
**Monitoring Frameworks**	General-purpose monitoring tools	Tools for lightweight network function instances, data collection from federated clouds and microservices, adoption of CNCF tools
**Identity Management & Accountability**	Centralized solutions, emerging distributed ledger technology, authentication based on public key and blockchains, digital identity across services	Multi-tenancy, encryption, privacy-preservation and scalability, accountability, support for protected services, consideration of computation offload and data portability
**Service Level Agreement Management**	Infrastructure-oriented perspective, user constraint at infrastructure-level, dependent on specific deployments	Automatic hybrid cloud SLA management, tailored negotiation for fog and cloud native deployments
**Anonymization and Encryption**	Lightweight and fine-grained data encryption and sharing, distributed access control, data privacy preservation	Anonymization for fine-grained techniques, legal and regulatory compliance, automated/on-demand hybrid cloud data privacy
**Fog Node Discovery**	Limited fog/edge discovery solutions, presence advertisement via broadcasting, cloudlet discovery through DNS-SD/Multicast DNS	Dynamicity and geo-distribution of edge devices, multiple networking domains, underlying technology abstraction, consideration of client constraints
**Hybrid Cloud Orchestration**	Cloud provider level federation, cross-cloud discovery, orchestration and scheduling for through Kubernetes, application portability across cloud infrastructures	Consideration of mostly static clusters and devices, automated federation lifecycle management, extensions to Kubernetes and its federation APIs
**Serverless Hybrid Data Lakes**	Centralized cloud-based solutions, serverless computations stacked as data analytic pipelines, introduction of specialized serverless orchestration systems	Cloud native SaaS, distributed serverless orchestration, portability using K8s, mix and match of computing paradigms via cloud native technologies

**Table 2:** Example use cases associated with expected benefits, costs and stakeholders.

Use Case	Benefits	Costs	Stakeholders
**Smart City Maintenance Scenario**	(1) enable real-time smart city maintenance services to city stakeholders; (2) trigger automated actions; (3) enable the flexible on demand deployment of data processing tasks.	IoT processing nodes located on busses and a central computation service in a data center, integrated within the smart city platform	Smart Cities applications developers; Transport company; Municipality; Cloud provider; Telecom operator
**Personalized Medicine Scenario**	(1) connect dispersed health data lakes ensuring security; (2) manage efficiently a federated platform hosting healthcare services; (3) enable new business models relying on the exploitation of health data and services across federated platforms.	Medical devices, wearables, remote sensors and wireless patches for patient status monitoring and data transmission to a gateway, edge and core cloud infrastructure.	Telecare providers; Hospitals; Insurance companies; Cloud provider; Telecom operator

## Abbreviations

The following abbreviations are used in this manuscript:

5G	Fifth Generation
AI	Artificial Intelligence
AIOTI	Alliance for the Internet of Things Innovation
APIs	Application Programming Interfaces
CNCF	Cloud Native Computing Foundation
DDoS	Distributed Denial of Service
DLT	Distributed Ledger Technology
DoS	Denial of Service
DPO	Data Pipeline Orchestrator
EaaS	Edge-as-a-Service
EHR	Electronic Health Records
FNs	Fog Nodes
GDPR	General Data Protection Regulation
IaaS	Infrastructure-as-a-Service
IoT	Internet of Things
MEC	Multi-access Edge Computing
OFRA	OpenFog Reference Architecture
OGF	Open Grid Forum
PaaS	Platform-as-a-Service
PPP	Public Private Partnerships
QoS	Quality of Service
SaaS	State-as-a-Service
SLA	Service Level Agreement
SME	Small and Medium-sized Enterprise
VM	Virtual Machine
XML	Extensible Markup Language
